# Identification of distinct molecular subtypes of uterine carcinosarcoma

**DOI:** 10.18632/oncotarget.15032

**Published:** 2017-02-02

**Authors:** Yang An, Haojie Wang, Jingyao Jie, Yitai Tang, Weijuan Zhang, Shaoping Ji, Xiangqian Guo

**Affiliations:** ^1^ Department of Biochemistry and Molecular Biology, Medical School, Henan University, Kaifeng 475004, China; ^2^ Cell Signal Transduction Laboratory, Henan University, Kaifeng 475004, China; ^3^ Department of Pathology, Stanford University School of Medicine, Stanford, CA 94305, USA; ^4^ Department of Oncology, The First Affiliated Hospital of Henan University, Kaifeng, 475001, China; ^5^ Department of Preventive Medicine, Medical School, Henan University, Kaifeng 475004, China; ^6^ Department of Burn and Plastic Surgery, The Affiliated Nanshi Hospital of Henan University, Nanyang, 473003, China

**Keywords:** uterine carcinosarcoma, molecular subtype, molecular signature, gene expression pattern, subtype-specific treatment

## Abstract

Uterine carcinosarcoma (UCS) is a rare but lethal neoplasm with high metastasis and recurrence rate, and to date, no molecular classification of UCS has been defined to achieve targeted therapies. In this study, we identified two distinct molecular subtypes of UCS with distinct gene expression patterns and clinicopathologic characteristics. Subtype I UCS recapitulates low-grade UCS, in contrast subtype II UCS represents high-grade UCS with higher tumor invasion rate and tumor weight. Interestingly, subtype I UCS is characterized by cell adhesion and apoptosis pathways, whereas genes over-expressed in subtype II UCS are more involved in myogenesis/muscle development. We also proposed certain potential subtype specific therapeutic targets, such as SYK (spleen tyrosine kinase) for subtype I and cell-cycle proteins for subtype II. Our findings provide a better recognition of UCS molecular subtypes and subtype specific oncogenesis mechanisms, and can help develop more specific targeted treatment options for these tumors.

## INTRODUCTION

Uterine carcinosarcoma (UCS), also named malignant mixed mullerian tumor (MMMT), is a malignant tumor [1] and is composed of carcinomatous and sarcomatous components [2]. Its carcinomatous component resembles high grade endometrioid, serous or clear cell endometrial carcinoma, thus UCS may be also considered as Type II endometrial carcinoma [2]. Although UCS is relatively rare, with an annual incidence rate at about 5.1-6.9 per 1,000,000 women [3], it is a dangerous, sometimes even lethal, form of tumor due to its high metastasis and relapse rate and highly complex pathological context [4, 5]. Treatment of UCS mainly depends on surgery, especially lymphadenectomy, which can greatly improve the overall survival rate of patients [4]. However, the five-year survival rate is still low (18%-39%) [6–9].

In the past decades, many malignant cancers (including bladder cancer, breast cancer, colon cancer, glioma, kidney cancer, leiomyosarcoma, ovarian cancer and prostate cancer) have been classified into different molecular subtypes on the basis of their different molecular signatures. Such classifications had helped better understand these tumors, and had contributed to the development of more targeted and effective therapies [10–19]. In this study, we describe the identification of two distinct molecular subtypes of uterine carcinosarcoma, each of which exhibits different gene expression patterns and clinicopathologic features. We also show that subtype II represents the high-grade UCS due to its high aggressiveness, and subtype II patients are less sensitive to the treatment than subtype I patients. Our study deepens our understanding of UCS and provides strategies to develop targeted therapies for UCS based on the different molecular subtypes.

## RESULTS

### Consensus clustering identifies two distinct molecular subtypes of UCS

**Figure 1:**
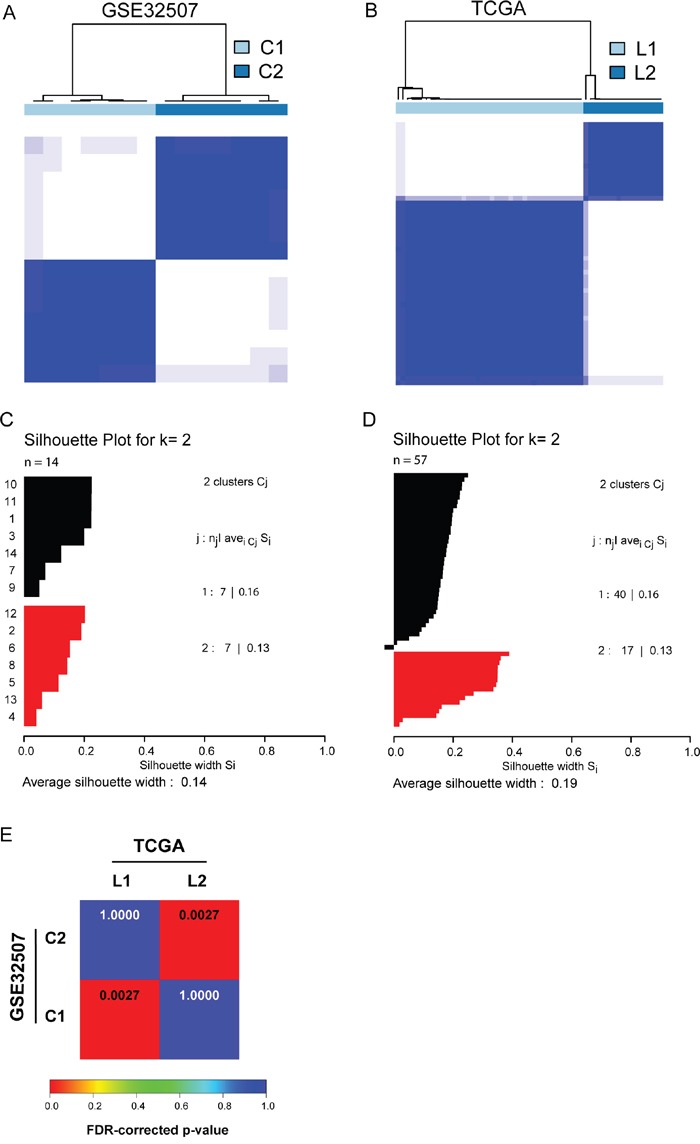
Identification of two molecular subtypes of UCS **A**. and **B**. Consensus clustering reveals two molecular subtypes of UCS in GSE32507 and TCGA dataset, respectively. **C**. and **D**. Silhouette analysis for GSE32507 and TCGA dataset validates the subtype assignments from consensus clustering, respectively. **E**. SubMap matrix showed significant correlation of subtypes from independent datasets. The correlation significance was denoted by FDR-corrected p-values.

We analyzed 14 cases of UCS obtained from Gene Expression Omnibus database (GSE32507), and identified two distinct molecular subtypes of UCS by consensus clustering (Figure 1A and 1C, two subtypes were designated C1 and C2). To expand the number of UCS cases in our study, we performed another independent analysis on a dataset with 57 UCS cases from The Cancer Genome Atlas (TCGA), the results of which also revealed that there are two distinct molecular subtypes of UCS (Figure 1B and 1D, two subtypes were designated L1 and L2). In both datasets, the optimal number of two subtypes was defined by consensus clustering, as indicated by the empirical cumulative distribution plots (Supplementary Figure 1A and 1C, Supplementary Figure 1B and 1D).

We subsequently performed a SubClass Mapping (SubMap) analysis to determine if the subtypes identified in the two above datasets are correlated. The SubMap result indeed showed that C1 and C2 subtypes in GSE32507 are highly correlated with L1 and L2 subtypes in TCGA dataset, respectively (Figure 1E).

These results suggest that there are two distinct molecular subtypes of UCS with different gene expression patterns.

### Clinicopathologic characteristics of UCS molecular subtypes

Next we compared the subtype specific clinicopathologic features of 57 UCS patients from TCGA dataset which provides more complete follow-up information of UCS patients (hereafter subtype L1 and L2 in TCGA dataset are designated as subtype I and subtype II, respectively, unless otherwise specified, Table 1 and Supplementary Table 1). As shown in Table 1, UCS mainly occurred in endometrium (58%), being histologically diagnosed as homologous and heterologous type (23% and 35%, respectively) or NOS (42%). It is noteworthy that almost half of subtype I patients (49%) were diagnosed as NOS, whereas more than half of subtype II patients (53%) were diagnosed as heterologous type UCS. Interestingly, different subtypes appear to correspond to different clinical grades, as subtype I is closely associated with stage I while subtype II UCS is more associated with stage III (χ^2^ test; *p*=0.03). The average percentage of tumor invasion of subtype I (36.22%) is significantly lower than that of subtype II (59.46), and the average tumor weight of subtype I (267.18) is also substantially lower than that of subtype II (vs 432.24). In contrast, the average percentage of tumor necrosis of subtype I (10.31%) is higher than that of subtype II (7.85%). And more subtype I patients than subtype II positively responded to treatment at the first course although it did not reach a significance (*p* = 0.09), indicating that subtype I patients may be more sensitive to treatment (Supplementary Table 1).

**Table 1:** Clinicopathologic Characteristics (N = 57)

Characteristic	patients, n(%)	Subtype I	Subtype II	Other UCS	p value
Age (year)					
Mean	70	70	69	72	
Range	51-90	51-90	60-88	72	
Tumor invasion percent					0.02*
Mean	45.01	36.22	59.46	83	
Range	0-100	0-100	8-100	83	
Grade					0.03*
Stage I	22 (38.5%)	20	2	0	
Stage II	5 (9%)	2	3	0	
Stage III	20 (35%)	11	9	0	
Stage IV	10 (17.5%)	6	3	1	
Hypertension					0.04*
Yes	28 (49%)	17	10	1	
No	24 (42%)	18	6	0	
Unknown	5 (9%)	4	1	0	
Tumor weight					0.05
Mean	317.29	267.18	432.24	Unknown	
Range	30-1735	30-907	150-1735	Unknown	
Histologic diagnosis					0.14
MMMT: Homologous Type	13 (23%)	10	3	0	
MMMT: Heterologous Type	20 (35%)	10	9	1	
MMMT: NOS	24 (42%)	19	5	0	
Location					0.30
Endometrium	33 (58%)	25	8	0	
Myometrium	1 (2%)	0	1	0	
Fundus Uteri	1 (2%)	1	0	0	
Isthmus uteri	1 (2%)	1	0	0	
Unknown	21 (37%)	12	8	1	

* p<0.05

The above clinical observations suggest that subtype I represents low-grade UCS with low tumor invasion rate and tumor weight, whereas subtype II represents high-grade UCS with high tumor invasion rate and tumor weight.

### Distinct molecular subtypes of UCS have different gene expression patterns

**Figure 2:**
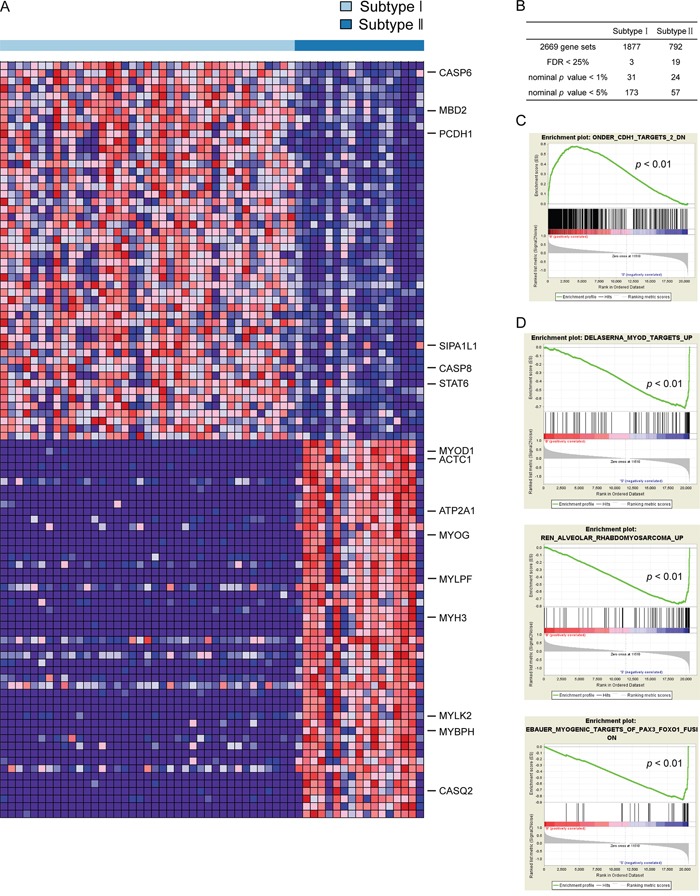
Different gene expression signatures enriched in distinct molecular subtypes **A**. Subtype I and subtype II have different gene expression patterns revealed by GSEA. Red, over-expressed genes; Blue, down-expressed genes. **B**. The summary of GSEA results. **C**. and **D**. The gene sets enriched in subtype I and subtype II reveal distinct gene expression signatures. Permutation=1000, *p*<0.01.

To further explore the subtype specific gene expression patterns for the two distinct subtypes of UCS, we performed Gene Set Enrichment Analysis (GSEA) [20]. As described above, the two molecular subtypes of UCS in TCGA dataset presented distinct gene expression patterns (Figure 2A). By analyzing 3396 gene sets with GSEA in TCGA dataset, 2669 gene sets were shown to be enriched in the two subtypes, with 1877 of them over-expressed in subtype I and the remaining 792 over-expressed in subtype II (Figure 2B). Interestingly, subtype II UCS is enriched with genes involved in myoblast differentiation/muscle development, such as *MYOD1* and *MYOGENIN*, which are the key factors in performing myogenic program (Figure 2A and 2C). While genes overexpressed in subtype I are associated with cell-cell adhesion and apoptosis, such as *PCDH1* (protocadherin 1), *CASP6* and *CASP8* (caspase 6 and 8) (Figure 2A and 2D).

### Different signatures and pathways are enriched in different molecular subtypes

We next investigated the genes showing significantly differential expression between two molecular subtypes of UCS in TCGA dataset by Significance Analysis of Microarrays (SAM-seq, two-class comparison). Among 2984 genes that were shown to have significant expression difference between two subtypes, 1206 genes are over-expressed in subtype I and down-expressed in subtype II UCS, in contrast, 1778 are over-expressed in subtype II and down-expressed in subtype I UCS. The Top500 over-expressed genes in each subtype were clustered, and those genes were shown to be significantly over-represented in subtype I and subtype II, respectively (Supplementary Figure 2). Then we performed Gene ontology (GO) and pathway analysis to identify the GO terms and pathways enriched in each subtype. Consistent with the GESA results, cell-cell adhesion and antigen processing and presentation pathways were shown to be enriched in subtype I, whereas muscle development and transcriptional activation pathways were found to be enriched in subtype II (Supplementary Table 2). Lastly, in order to identify potential therapeutic targets for each UCS molecular subtype, we compared the genes specifically over-expressed in each UCS molecular subtype with genes involved by activating mutations or amplifications from TARGET database (tumor alterations relevant for genomics-driven therapy) (https://www.broadinstitute.org/cancer/cga/target), the database which includes gene-targeted therapeutic methods currently available in clinics or under development. This would allow us to take advantage of the currently available therapeutic targets to develop more targeted or precision UCS therapies. 14 significantly over-expressed genes in subtype I UCS were annotated as potential therapeutic targets, such as SYK (spleen tyrosine kinase). In contrast, 12 significantly over-expressed genes in subtype II UCS were annotated as potential therapeutic targets, including CCNE1 (Cyclin E1), CCND2 (Cyclin D2) and CDK6 (Cyclin dependent kinase 6) (Table 2).

**Table 2:** Target genes enriched in each molecular subtype

	Gene Over-expressed	Examples of Potential Therapeutic Agents
Subtype I	SYK	SYK inhibitors
	CRKL	Gefitinib, Erlotinib, EGFR inhibitorsVemurafenib, Dabrafenib, RAF inhibitorsDasatinib, SRC inhibitors
	CCNE1	CDK2 inhibitor
Subtype II	CDK6	CDK4/6 inhibitors
	NOTCH2	Notch Inhibitors
	CCND2	CDK4/6 inhibitors
	CCND3	CDK4/6 inhibitors

## DISCUSSION

Uterine carcinosarcoma (UCS) is a rare malignant tumor, making up less than 5% of uterine neoplasm, but contributing to approximately 30% uterine cancer mortality due to its high metastasis rate [3]. As the term suggests, UCS is a biphasic neoplasm that contains both carcinoma and sarcoma components. Based on the origin of sarcomatous component, there are two types of UCS: heterologous-type and homologous-type [21]. The heterologous-type is composed of components derived from skeletal muscle, cartilage or bone, whereas the sarcoma component in homologous-type is from endometrium [1]. In the past decades, it has been reported that the heterologous-type UCS is more aggressive and those patients show poorer prognosis [22, 23]. In agreement with that, a big portion of patients classified as subtype II in our study were previously diagnosed as heterologous-type UCS (Table 1).

UCS can be categorized into four stages: stage I tumors are limited in the corpus uterus, stage II tumors infiltrate the cervical stroma but still being confined to the uterus, stage III tumors metastasize to proximal tissues such as para-aorticlymph nodes, and stage IV tumors metastasize to extra-pelvic such as bladder and bowel mucosa [1, 5]. Here we found that more than half of subtype I patients are likely to be at stage I and possesses the characteristics of less tumor invasion and low tumor weight (Table 1). In contrast, 53% of subtype II patients are likely to be at stage III and exhibit high metastasis rate and high tumor weight (Table 1).

Genes and pathways over-expressed in subtype I include those involved in cell-cell adhesion, cell apoptosis, lipid biosynthetic and metabolic process (Figure 2A and 2D, Supplementary Table 2). For example, *PCDH1*, a gene that belongs to the cadherin superfamily and mediates cell-cell adhesion activity [24], was found to be significantly over-expressed in subtype I UCS. *CASP8* and *CASP6*, two caspase family members that are responsible for the initiation and execution of apoptosis, were also found to be highly expressed in subtype I UCS [25].

The pathways enriched in subtype II include muscle development and contraction, macromolecule biosynthetic and metabolic process, transcription and nucleic acid metabolic process (Figure 2A and 2C, Supplementary Table 2). *MYOD1* and *MYOGENIN* which were reported to be key myogenic regulatory factors driving myoblast differentiation [26], were over-expressed in subtype II UCS. This suggests that muscle infiltration may be involved in the development of UCS microenvironment [27, 28], or UCS tumor cells may exhibit the muscle differentiation molecular characteristics [29, 30].

At present, the optimal therapeutic regimen is still under discussion [31]. Compared to uterine sarcoma or endometrial carcinoma, UCS is more difficult to treat due to its complex pathologic context, metastasis to lymph nodes and high relapse rate [4, 23, 32, 33]. That might explain why UCS has higher mortality rate than other uterine tumors although it is relatively rare. Therefore, if UCS patients can be classified into distinct molecular subtypes, therapies targeting specific subtypes will likely offer better clinical benefits.

Remarkably, in our study, subtype I patients are more sensitive to treatment than subtype II patients at the first course, and this finding may guide the future treatment of UCS patients. It is noteworthy that SYK, an oncogenic kinase and a potential therapeutic target for Small-cell lung cancer and hematologic neoplasms [34–36], was over-expressed in subtype I (Table 2). Inhibition of SYK activity by SYK-specific inhibitors such as PRT060318 and fostamatinib disodium [37, 38] provides great clinical outcome to certain cancer patients with abnormal SYK activities, indicating that subtype I patients may be benefited from SYK-specific kinase inhibitors.

Subtype II exhibited high expression level of *CRKL*, *CCNE1*, *CDK6*, *NOTCH2*, *CCND2* and *CCND3* (Table 2). Among those, CDK6, CCND2, CCND3 and CCNE1 are mitotic cell cycle-related proteins and inhibited by CDK4/6 inhibitors and CDK2 inhibitor, respectively. Abemaciclib, palbociclib and ribociclib are CDK4/6 inhibitors and have been used to treat various cancers, including breast cancer, colorectal cancer, glioblastoma, liposarcoma, melanoma, non-small cell lung cancer and hematologic malignancies [39–41]. CCNE1 is another therapy target, and it is found to be over-expressed in endometrial carcinomas [42], bladder carcinoma [43], ovarian cancer [44] and non-BRCAness high grade ovarian carcinoma [45]. Cancer patients with high *CCNE1* expression levels have shown increased sensitivity to CDK2 inhibitors SNS-032 [46]. Therefore, inhibitors targeting cell-cycle proteins may potentially provide better therapeutic effects to subtype II patients.

In summary, our findings provide new insight into the intrinsic molecular stratification in UCS and particular mechanism underlying tumorigenesis and tumor progression, making it possible that the future targeted treatment of UCS is performed in a subtype-specific manner, and finally help to guide the precision medicine for UCS patients.

## MATERIALS AND METHODS

### Bioinformatic analyses

To identify the molecular subtypes of UCS, we analyzed expression profile data of GSE32507 and TCGA datasets by consensus clustering and SubMap. After filtering the whole expression dataset with standard deviation and adjusting the filtered dataset with gene-based centering, the optimal number of molecular subtypes of UCS cases was determined by Consensus Clustering (R package ConsensusClusteringPlus [47], with parameters of distance (1-Pearson correlation), 80% sample resampling, 80% gene resampling, maximum evaluated k of 12, and agglomerative hierarchical clustering algorithm over 1000 iterations). Silhouette analysis (R package cluster [48]) was then used to evaluate the accuracy of subtype assignments from ConsensusClusteringPlus. Gene Set Enrichment Analysis (GSEA) and Significance Analysis of Microarrays (SAM-seq) were used to investigate the subtype specific gene expression patterns and pathways. The Gene ontology analysis was applied to identify the pathways enriched in each subtype by the Database for Annotation, Visualization and Integrated Discovery [24] online (https://david.ncifcrf.gov/). Cluster 3.0 and TreeView were used to do hierarchical clustering to view TOP500 significantly over-expressed genes from each subtype. The target genes enriched in each subtype were explored by comparing SAM-seq result with data from TARGET (tumor alterations relevant for genomics-driven therapy) database.

### Statistical analyses

The significance was assessed by the chi-square and Fisher exact tests and *p* value less than 0.05 was considered significant.

## SUPPLEMENTARY MATERIALS FIGURES AND TABLES




