# Supplementary material for: Identification of distinct molecular subtypes of uterine carcinosarcoma
Source: Oncotarget. 2017 Feb 2;8(9):15878–86. doi: 10.18632/oncotarget.15032 (PMC5362530; doi:10.18632/oncotarget.15032)
Supplement: Supplementary file 1 [file oncotarget-08-15878-s001.pdf]

Identification of distinct molecular subtypes of uterine carcinosarcoma

SUPPLEMENTARY FIGURES AND TABLES

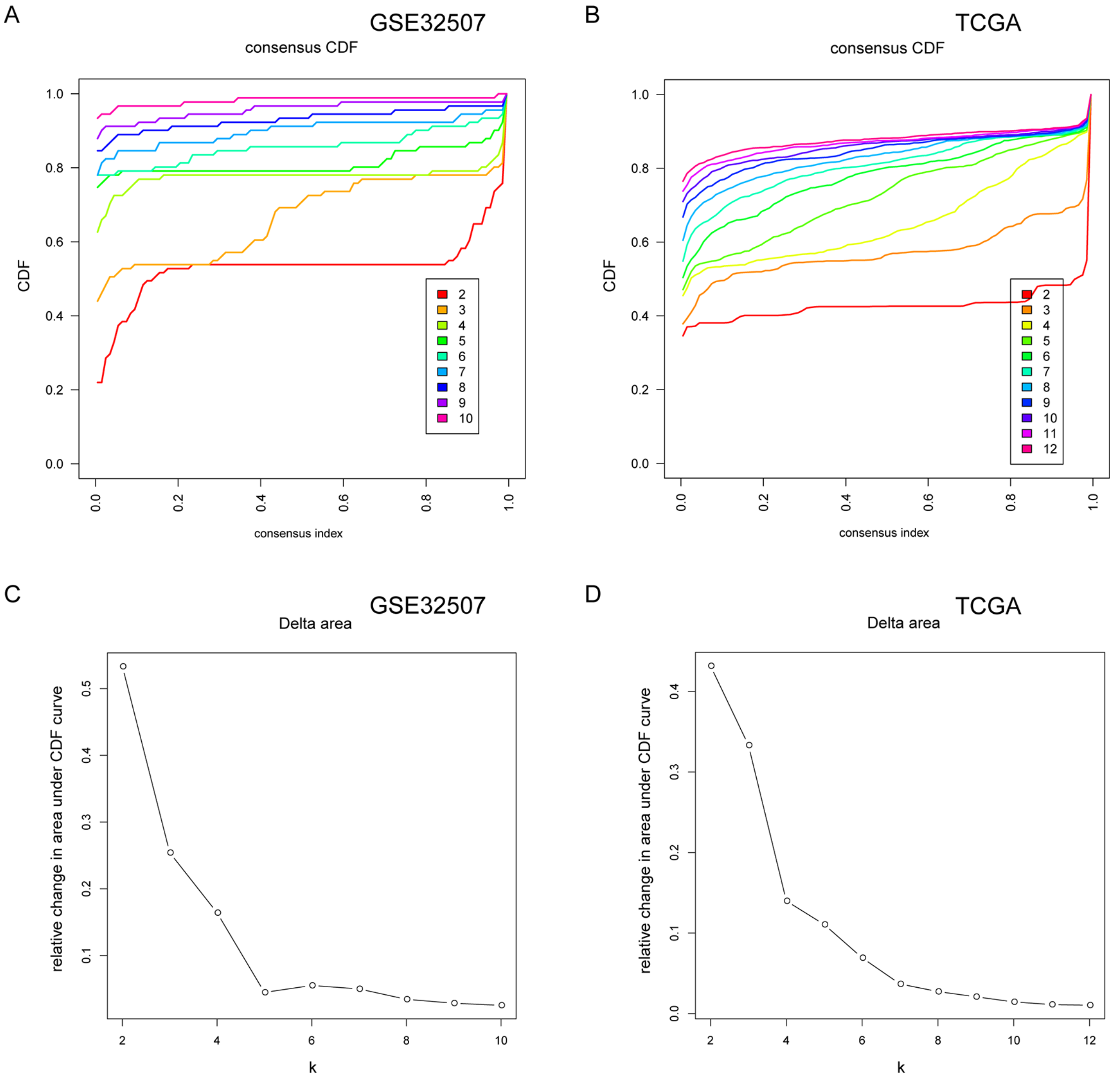

Supplementary Figure 1: Delineation of two molecular subtypes of UCS. A. and **B.** Empirical cumulative distribution plots for GSE32507 and TCGA dataset, respectively. **C.** and **D.** The increased area under the CDF curve along with increased number of molecular subtypes.

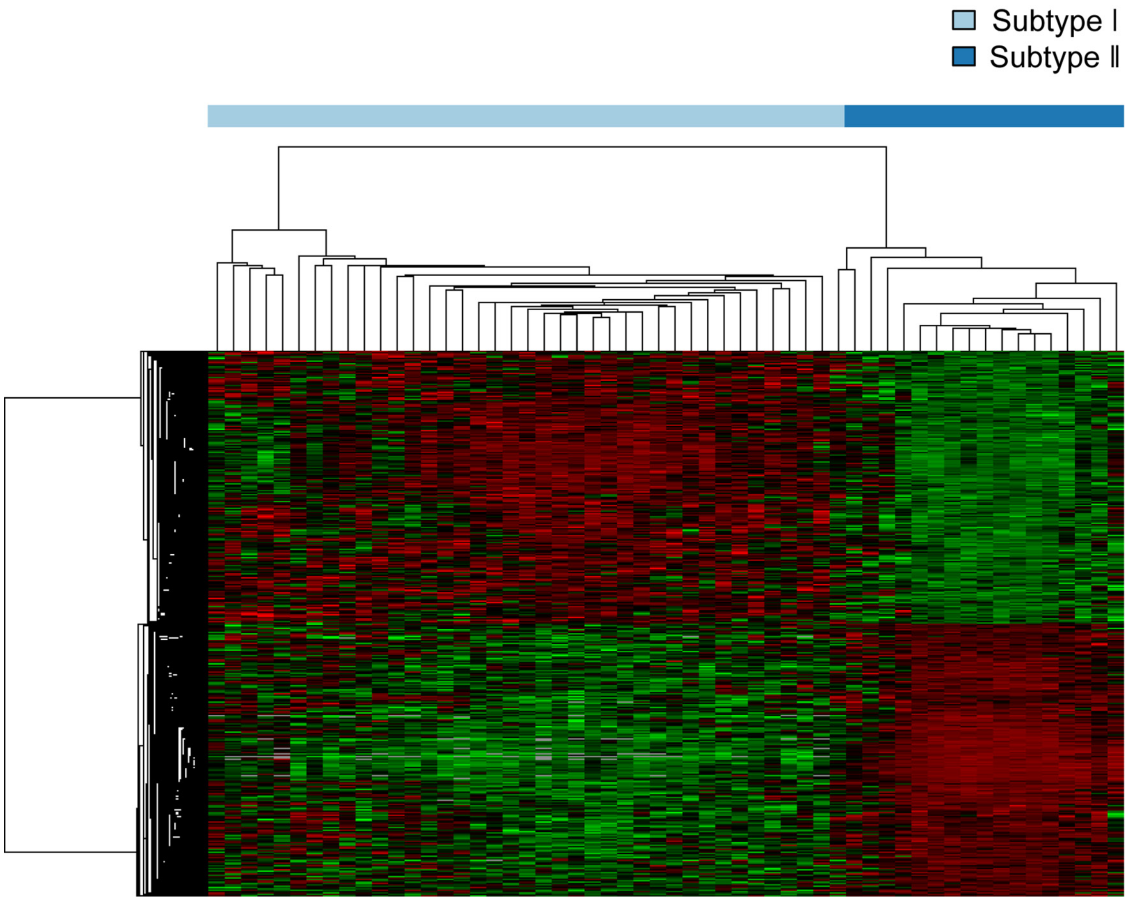

Supplementary Figure 2: Heatmap of top 500 genes over-expressed in distinct molecular subtypes. After SAM-seq, we picked top 500 genes over-expressed in each subtype to form TOP500. Hierarchical clustering of TOP500 was performed by Cluster 3.0 using centroid linkage method. Red, over-expressed genes; Green, down-expressed genes.

Supplementary Table 1: Clinicopathologic Characteristics (N = 57)

See Supplementary File 1

Supplementary Table 2: Pathways enriched in each molecular subtype

See Supplementary File 1
