# Supplementary material for: Identification of distinct molecular subtypes of uterine carcinosarcoma
Source: Oncotarget. 2017 Feb 2;8(9):15878–86. doi: 10.18632/oncotarget.15032 (PMC5362530; doi:10.18632/oncotarget.15032)
Supplement: Supplementary file 2 [file oncotarget-08-15878-s002.docx]

**Supplementary Tables**

**Table S1**. Clinicopathologic Characteristics (*N* = 57)

| Characteristic | patients, *n*(%) | SubtypeⅠ | SubtypeⅡ | Other UCS | *p* value |
| --- | --- | --- | --- | --- | --- |
| Sex |  |  |  |  |  |
| Female | 57 (100%) | 39 | 17 | 1 |  |
| Male | 0 | 0 | 0 | 0 |  |
| Tumor nuclei percent |  |  |  |  | 0.05 |
| Mean | 80.63 | 78.26 | 87 | Unknown |  |
| Range | 25-100 | 25-100 | 70-98 | Unknown |  |
| Treatment outcome first course | |  |  |  | 0.09 |
| Response | 34 (60%) | 26 | 7 | 1 |  |
| Non-response | 13 (23%) | 7 | 6 | 0 |  |
| Unknown | 3 (5%) | 2 | 1 | 0 |  |
| Not Available | 5 (9%) | 4 | 1 | 0 |  |
| Not Applicable | 2 (4%) | 0 | 2 | 0 |  |
| Lymph nodes pelvic examined count | |  |  |  | 0.10 |
| Mean | 10.02 | 11.54 | 7 | 2 |  |
| Range | 0-34 | 0-34 | 1-26 | 2 |  |
| Weight (kg) |  |  |  |  | 0.20 |
| Mean | 73.31 | 75.97 | 68.06 | 64 |  |
| Range | 42-167 | 44-167 | 42-88 | 64 |  |
| Diabetes |  |  |  |  | 0.20 |
| Yes | 6 (11%) | 3 | 3 | 0 |  |
| No | 45 (79%) | 33 | 11 | 1 |  |
| Unknown | 6 (11%) | 3 | 3 | 0 |  |
| History other malignancy |  |  |  |  | 0.24 |
| Yes | 8 (14%) | 7 | 1 | 0 |  |
| No | 49 (86%) | 32 | 16 | 1 |  |
| Unknown | 0 | 0 | 0 | 0 |  |
| Percent lymphocyte infiltration | |  |  |  | 0.27 |
| Mean | 18.28 | 16.38 | 23.71 | 0 |  |
| Range | 0-70 | 0-70 | 0-70 | 0 |  |
| Race |  |  |  |  | 0.35 |
| White | 44 (77%) | 30 | 13 | 1 |  |
| Black or African American | 9 (16%) | 5 | 4 | 0 |  |
| Asian | 3 (5%) | 3 | 0 | 0 |  |
| Unknown | 1 (2%) | 1 | 0 | 0 |  |
| Percent neutrophil infiltration |  |  |  |  | 0.39 |
| Mean | 8.86 | 10 | 6.76 | 0 |  |
| Range | 0-40 | 0-40 | 0-20 | 0 |  |
| Percent normal cells |  |  |  |  | 0.39 |
| Mean | 1.58 | 1.15 | 2.64 | 0 |  |
| Range | 0-30 | 0-30 | 0-30 | 0 |  |
| Percent monocyte infiltration |  |  |  |  | 0.53 |
| Mean | 7.56 | 7.18 | 8.88 | 0 |  |
| Range | 0-20 | 0-20 | 0-20 | 0 |  |
| Tumor necrosis percent |  |  |  |  | 0.59 |
| Mean | 9.65 | 10.31 | 7.85 | Unknown |  |
| Range | 0-85 | 0-85 | 0-20 | Unknown |  |
| Percent tumor cells |  |  |  |  | 0.59 |
| Mean | 87.12 | 87.72 | 85.88 | 85 |  |
| Range | 60-100 | 60-100 | 60-100 | 85 |  |
| Percent stromal cells |  |  |  |  | 0.71 |
| Mean | 5.79 | 6.03 | 5.29 | 5 |  |
| Range | 0-20 | 0-20 | 0-20 | 5 |  |
| Height (cm) |  |  |  |  | 0.92 |
| Mean | 157.71 | 157.8 | 157.56 | 157 |  |
| Range | 138-172 | 138-172 | 140-170 | 157 |  |
| pharmaceutical tx adjuvant |  |  |  |  | 0.95 |
| Yes | 36 (63%) | 25 | 10 | 1 |  |
| No | 18 (32%) | 13 | 5 | 0 |  |
| Discrepancy | 1 (2%) | 1 | 0 | 0 |  |
| Unknown | 2 (4%) | 0 | 2 | 0 |  |
| radiation treatment adjuvant |  |  |  |  | 0.96 |
| Yes | 25 (44%) | 18 | 7 | 0 |  |
| No | 29 (51%) | 20 | 8 | 1 |  |
| Discrepancy | 1 (2%) | 1 | 0 | 0 |  |
| Unknown | 2 (4%) | 0 | 2 | 0 |  |

**Table S2**. Pathways enriched in each molecular subtype.

| Category | Pathways | *p* Value | |
| --- | --- | --- | --- |
|  |  | SubtypeⅡ | SubtypeⅠ |
| Muscle development | muscle system process | 8.59E-32 |  |
|  | striated muscle tissue development | 6.88E-15 |  |
|  | muscle tissue development | 9.46E-15 |  |
|  | skeletal muscle tissue development | 8.44E-12 |  |
|  | muscle cell differentiation | 6.90E-10 |  |
|  | striated muscle cell differentiation | 6.62E-08 |  |
| Muscle contraction | muscle contraction | 3.29399E-33 |  |
|  | striated muscle contraction | 2.08115E-20 |  |
|  | regulation of muscle contraction | 1.51552E-06 | 0.083899117 |
|  | regulation of striated muscle contraction | 1.12E-05 |  |
|  | skeletal muscle contraction | 3.19E-05 |  |
| Cytoskeleton organization | cytoskeleton organization | 1.65E-05 | 0.017621582 |
|  | actin filament-based process | 2.52E-05 | 0.00275735 |
|  | actin cytoskeleton organization | 2.60E-04 | 0.002268395 |
|  | actin filament-based movement | 7.79E-04 |  |
| Pattern specification | anterior/posterior pattern formation | 3.61E-09 |  |
|  | pattern specification process | 5.31E-09 |  |
|  | cell fate commitment | 1.22E-06 |  |
| Heart development & contraction | heart development | 6.59E-08 |  |
|  | regulation of heart contraction | 6.80E-08 |  |
|  | cardiac muscle contraction | 2.07E-06 |  |
|  | cardiac muscle tissue development | 4.45E-05 |  |
| Morphogenesis | tissue morphogenesis | 5.79E-09 |  |
|  | embryonic morphogenesis | 1.70E-06 | 0.078541673 |
|  | embryonic organ morphogenesis | 2.21E-04 | 0.041800605 |
| Biosynthesis | positive regulation of transcription | 3.28E-05 |  |
|  | positive regulation of macromolecule biosynthetic process | 3.95E-05 |  |
|  | positive regulation of gene expression | 5.47E-05 |  |
| Metabolism | positive regulation of nucleobase, nucleoside, nucleotide and nucleic acid metabolic process | 1.04E-05 |  |
|  | positive regulation of macromolecule metabolic process | 2.10E-04 |  |
|  | positive regulation of RNA metabolic process | 9.47E-04 |  |
|  | membrane lipid metabolic process |  | 1.47E-04 |
|  | sphingolipid metabolic process |  | 5.89E-05 |
|  | lipid biosynthetic process |  | 6.56E-04 |
| Ion transport | calcium ion transport | 1.99E-06 |  |
|  | metal ion transport | 1.19E-05 |  |
| Cell adhesion | cell adhesion |  | 2.45E-04 |
|  | biological adhesion |  | 2.54E-04 |
|  | cell-cell adhesion |  | 0.002621085 |
|  | regulation of cell-cell adhesion |  | 0.010703341 |
| Cell motion | cell motion |  | 1.42E-04 |
|  | cell motility |  | 0.006692312 |
|  | cell migration | 0.084442544 | 0.004966052 |
| Epithelium development | epithelium development | 0.078520627 | 2.29E-06 |
|  | epithelial cell differentiation |  | 4.71E-05 |
| Ectoderm development | ectoderm development |  | 5.60E-05 |
| Epidermis development | epidermis development |  | 9.50E-05 |
| Antigen processing and presentation | antigen processing and presentation of peptide antigen via MHC class I |  | 4.40E-05 |
|  | antigen processing and presentation of peptide antigen |  | 2.48E-04 |
|  | antigen processing and presentation |  | 0.002033974 |
| Leukocyte | leukocyte migration |  | 2.79E-03 |
|  | leukocyte chemotaxis |  | 7.68E-03 |
|  | leukocyte activation |  | 1.07E-02 |
| Signal transduction | regulation of small GTPase mediated signal transduction | | 2.87E-04 |
|  | integrin-mediated signaling pathway |  | 3.90E-04 |
|  | regulation of Ras protein signal transduction |  | 0.001706518 |
| Kinase activity | positive regulation of kinase activity | 0.002891894 | 7.02E-04 |
|  | positive regulation of protein kinase activity | 0.001579674 | 8.82E-04 |
|  | activation of protein kinase activity | 3.10E-04 | 0.005418386 |
